# Outcomes of 42 pregnancies in 14 women with cartilage-hair hypoplasia: a retrospective cohort study

**DOI:** 10.1186/s13023-020-01614-2

**Published:** 2020-11-19

**Authors:** Elina Holopainen, Svetlana Vakkilainen, Outi Mäkitie

**Affiliations:** 1 Department of Reproductive Medicine, Obstetrics and Gynecology, Helsinki University Hospital and University of Helsinki, P.O. Box 140 00029, Helsinki, Finland; 2 Research Program for Clinical and Molecular Metabolism, University of Helsinki, Helsinki, Finland; 3 Children’s Hospital, University of Helsinki and Helsinki University Hospital, Helsinki, Finland; 4 Folkhälsan Research Center, Helsinki, Finland; 5 Department of Molecular Medicine and Surgery, Karolinska Institutet and Department of Clinical Genetics, Karolinska University Hospital, Stockholm, Sweden, Karolinska Institutet and Karolinska University Hospital, Stockholm, Sweden

**Keywords:** Pregnancy, Cartilage-hair hypoplasia, Obstetric, Cesarean section, Miscarriage

## Abstract

**Background:**

Cartilage-hair hypoplasia (CHH) is a rare skeletal dysplasia characterized by disproportionate short stature, immunodeficiency, anemia and risk of malignancies. All these features can affect pregnancy and predispose to maternal and fetal complications. This study aimed to evaluate obstetric history and maternal and fetal outcomes in women with CHH.

**Methods:**

Among 47 Finnish women with CHH, we identified 14 women with ICD codes related to pregnancies, childbirth and puerperium in the National Hospital Discharge Registry and obtained detailed data on gynecologic and obstetric history with a questionnaire. Offspring birth length and weight were collected and compared with population-based normal values.

**Results:**

There were altogether 42 pregnancies in 14 women (median height 124 cm, range 105–139 cm; 4′1′′, range 3′5′′–4′7′’). Twenty-six pregnancies (62%), including one twin pregnancy, led to a delivery. Miscarriages, induced abortions and ectopic pregnancies complicated 9, 5, and 2 pregnancies, respectively. Severe pregnancy-related complications were rare. All women with CHH delivered by cesarean section, mostly due to evident cephalo-pelvic disproportion, and in 25/26 cases at full-term. In the majority, the birth length (median 48 cm, range 45.5–50 cm; 1′7′′, range 1′6′′–1′8′′) and weight (3010 g, range 2100–3320 g; 6.6 lb, range 4.6–7.3 lb) of the offspring in full-term singleton pregnancies was normal.

**Conclusions:**

Despite CHH mothers’ significant short stature and other potential CHH-related effects on pregnancy outcome, most pregnancies lead to a term cesarean section delivery. Since fetal growth was generally unaffected, cephalo-pelvic disproportion was evident and planned cesarean section should be contemplated in term pregnancies.

## Background

Bone dysplasia, small maternal height and disproportion can all affect fertility and natural course of pregnancy, and may predispose to pregnancy and obstetric complications affecting perinatal outcomes. Women with short stature may suffer from respiratory distress during pregnancy and experience more cephalo-pelvic disproportion (CPD) leading to high rate of cesarean deliveries (CS) [1, 2].

Some studies have previously been performed on maternal and fetal outcome in different bone dysplasias. Pregnancies in patients with osteogenesis imperfecta can be complicated with antepartum hemorrhage, placenta abruption, intrauterine growth restriction (IUGR) and the birth of small-for-gestational-age infants [3]. Pregnancy-related complications in women with achondroplasia are uncommon, the most serious complications being worsening of spinal claudication symptoms and respiratory failure. Most women with achondroplasia have term pregnancies, but planned CS is required because of CPD [4]. However, most obstetric reports in patients with short stature due to skeletal dysplasia are case reports and have mainly focused on anesthetic planning and performance of cesarean delivery [5, 6].

Cartilage-hair hypoplasia (CHH) is a rare autosomal recessive disorder with an incidence of 1: 23,000 live births in Finland [7]. It is characterized by short stature, hypoplastic hair, combined immunodeficiency and increased risk for malignancies, especially lymphoma. CHH is caused by variants in the *RMRP* gene, encoding the RNA subunit of the mitochondrial RNA processing endonuclease, which is involved in cell cycle regulation [8]. CHH is a disease with significant variability in phenotypic presentation [9]. Women with CHH have adult height in the range of 110–140 cm (3′7′′–4′7′′), median height being 122.5 cm (4′0′′) [10].

Disproportionate short stature and immunodeficiency are among the most prominent features of CHH and can adversely influence female patients’ reproductive health. Immunodeficiency can predispose pregnant women to infections during naturally immunocompromised state in pregnancy. In addition to maternal complications, short stature might affect fetal growth and predispose to preterm deliveries. Despite the growing knowledge of disease mechanism, many clinical aspects of CHH remain uncharacterized. Only limited data are available regarding puberty, reproduction, and gynecologic health in patients with CHH [11–13]. The obstetrical literature in CHH is limited to case reports [14, 15].

The knowledge gaps in reproductive and obstetric outcomes are a significant source of concern for patients and families affected by CHH. Lack of knowledge may affect CHH patients’ family planning. The possibility of obstetric complications can lead medical personnel to discourage the patient from becoming pregnant.

The purpose of this study was to evaluate the incidence and course of pregnancies and maternal and perinatal outcomes in the Finnish cohort of women with CHH.

## Patients and methods

### Patients

Patients were identified from the Finnish Skeletal Dysplasia Registry which includes > 110 living patients with genetically confirmed CHH; 56 of them were women aged over 18 years. Altogether 47 of these women were participants in our ongoing research program on CHH. Their ICD-10 codes related to pregnancies, childbirth and puerperium (O00*-O99*) were searched from the National Hospital Discharge Registry (HILMO). Of the 47 women with CHH, 19 had pregnancy or delivery-related diagnoses in the Registry, involving a total of 57 pregnancies. These 19 women were subsequently invited to participate in a questionnaire study of gynecologic and obstetric history; 14 consented. Thus, our final study population included 14 CHH women with 42 pregnancies.

### Methods

All 14 patients were either interviewed by one of the authors (EH) for data regarding reproductive and obstetric history (n = 10) or they provided the information using a specific questionnaire (n = 4). The interview and questionnaire covered pregnancy-related issues such as the number and outcome of pregnancies, pregnancy complications, pregnancy weeks (pw) at delivery, mode of delivery, delivery and post-partum complications and birth weight (BW), birth length (BL) and Apgar score of the newborns. Medical records of maternal outcomes were reviewed for all women, whereas offspring’s medical records were obtained only with a separate permission given by 9 of the 14 women and involving 19 children.

BWs and BLs of the offspring were compared with the Finnish growth references [16] and transformed into Z-scores for all newborns. We excluded from further growth analysis a pair of twins and one child born prematurely at 25 pw with IUGR.

An ethical approval was obtained from the Research Ethics Committee of the Hospital District of Helsinki and Uusimaa (HUS/836/2018 Institutional Research Ethics Committee).

### Mutation analysis

All *RMRP* mutations had been detected by Sanger sequencing either at Laboratory HUSLAB, Finland, or as a part of previous or ongoing research at Folkhälsan Institute of Genetics, Helsinki [17, 18].

## Results

### Cohort characteristics

The study cohort consisted of 14 CHH women with 42 pregnancies (Table 1). At data collection, the median age of the cohort was 45.0 years (range 31–72 years). All patients had genetically confirmed *RMRP* mutation NR_003051.3: n.71A > G in either homozygous (n = 11) or compound heterozygous (n = 3) state. The median height of the cohort was 124 cm, range 105–139 cm (4′1′′, range 3′5′′–4′7′′).

**Table 1:** Type of 42 pregnancies in 14 women with CHH

Type of pregnancy	N (% of all 42 pregnancies)	Maternal age (median)	Maternal age (range)
Early miscarriage (< 12 pw^a^)	8 (19)	34^b^	23-40^b^
Late miscarriage (< 22 pw)	1 (2)		
Ectopic pregnancy	2 (5)	34.5	34–35
Induced abortion	5 (12)	36	23–39
Term delivery (37–42 pw) including one twin delivery	25 (60)	32^c^	24-45^c^
Preterm delivery (< 37 pw)	1 (2)		

^a^pw, pregnancy weeks

^b^Combined data from all miscarriages

^c^Combined data from all pregnancies leading to delivery

According to the clinical categorization of immunodeficiency in CHH, 6/14 females had clinical symptoms of immunodeficiency in adulthood, mostly recurrent rhinosinusitis. Three of the six were classified as having clinical combined immunodeficiency because of opportunistic infections like severe herpes virus infections or recalcitrant warts [19].

### Miscarriages, ectopic pregnancies and induced abortions

Miscarriages (n = 9) were reported by 7 women. Eight miscarriages occurred during first trimester. None of the women had recurrent miscarriage, defined as loss of three or more consecutive pregnancies. Ectopic pregnancies occurred in two women. However, 89% (8/9) had also a successful pregnancy leading to a delivery.

Five induced abortions were reported in four women. Three were induced because of social indications and two because of fetal indications other than CHH.

### Deliveries

A total of 27 live births, including one pair of twins, were reported in 12 women. Maternal characteristics in pregnancies leading to delivery are presented in Table 2. Median maternal age at delivery was 32 years (range 24–45 years). Twenty-six (96%) of the children were born at full-term (pw 37 + 0—41 + 6). One preterm delivery due to fetal indication at pw 25 was reported.

**Table 2:** Maternal characteristics (n = 12) in 26 pregnancies leading to delivery

Characteristics	Median	Range
Age (years)	32	24–45
Height (cm / ft-inch)	124; 4′11′	105–139; 3′5′’–4′7′
BMI before pregnancy (kg/m^2^)^a^	33.5	29.4–59
Weight gain during pregnancy (kg; lb)^b^	8.4; 18.5	2.7–12.6; 5.6–27.8

^a^Data available from 21 pregnancies

^b^Data available from 16 pregnancies

All infants were delivered by CS. Two trials of vaginal labor, one induced and one spontaneous, were converted to CS, one of them complicated by chorioamnionitis. Indications for CS are presented in Table 3. Malpresentation was reported in six cases: fetus was either in breech or occiput posterior presentation in four and two cases, respectively. Two women had had two CSs, and three and two women had had three and four CSs, respectively. In four CSs, delivery of the baby was difficult and in two cases vacuum-extraction was required also in CS. Successful epidural anesthesia was difficult to accomplish potentially due to disproportionate spinal anatomy in 3/25 women and they reported intraoperative pain.

**Table 3:** Indication and type of cesarean section in 26 deliveries

Indication for CS^a^	N	Type of CS planned/emergency
CPD^b,c^	9	(1 Emergency, 8 planned)
Previous CS	9	Planned
CPD and previous CS	4	Planned
Twin pregnancy and precious CS	1	Planned
Unsuccessful labor induction and IUGR^d^	1	Emergency
IUGR and asphyxia	1	Emergency
Arrest of labor and fetal position	1	Emergency

^a^*CS* cesarean section

^b^*CPD* cephalo-pelvic disproportion

^c^In two cases vacuum extraction required

^d^*IUGR* intrauterine growth restriction

### Maternal complications during pregnancy

Ten of the 12 women with a history of delivery (83%) reported one or several pregnancy-related complications (Table 4). Most of the complications were sporadic and did not require hospitalization. There was no severe maternal morbidity. Despite underlying immunodeficiency and increased susceptibility to infections, no infections requiring hospitalization during pregnancy were reported. Anemia was reported in four pregnancies (15%) but none of them required blood transfusions or hospitalization. Two cases of placental insufficiency leading to fetal distress were diagnosed (2/27, 7%) based on elevated resistance or retrograde flow of *a. umbilicalis* and IUGR or fetal heart rate abnormalities and IUGR. No other placenta-related maternal complications, including severe pre-eclampsia or placental abruption, were reported.

**Table 4:** Maternal disorders predominantly related to pregnancy in 26 pregnancies leading to delivery

	N	% of 26 pregnancies
Maternal disorders related to pregnancy		
Anemia^a^	4	15
Hypertension^b^	3	12
Gestational diabetes^c^	3	12
Placental insufficiency	2	8
Ischial pain	1	4
Chorioamnionitis	1	4
Subjective respiratory distress (3rd trimester)	1	4
First-trimester bleeding	1	4
Hyperemesis	1	4
Post partum complications		
Cesarean section wound infection	1	4
Other post-partum infection	2	8

^a^Macrocytic anemia in 3 cases, iron deficiency 1 case; none required blood cell transfusions

^b^Blood pressure (BP) > 140/90 mmHg or systolic BP increase > 30 mmHg or diastolic BP increase > 15 mmHg during pregnancy

^c^Diet or insulin controlled

### Offspring’s birth characteristics

Birth measurements were assessed in 19 offspring for nine women. We calculated Z-scores for BL and BW based on Finnish references for healthy children. We excluded a pair of twins and one child born prematurely (at 25 pw) from further analysis. In the remaining neonates (n = 16), both BL and BW were in the majority below the normal mean (for BL median Z-score − 0.4, for BW median Z-score − 0.6) (Table 5). However, both measurements remained within the normal distribution (Z-score > − 2.0), except for one newborn whose BL Z-score was -2.4 and BW Z-score -3.3.

**Table 5:** Offspring’s birth characteristics (n = 16)

Characteristic	Median	Range
Pregnancy week	38 + 4	37 + 2—41 + 3
Birth weight (g, lb)	3010; 6.64	2100–3320; 4.63–7.32
Birth weight Z-score	− 0.6	− 3.3 − 0.0
Birth length (cm, ft-inch)	48; 1′7′′	45.5–50; 1′6′′–1′8′′
Birth length (Z-score)	− 0.4	− 2.4—+ 0.7
Apgar score (5 min)	9	4–10

One pair of twins and one preterm baby were excluded from the analysis

## Discussion

Pregnant women with skeletal dysplasia are at an increased risk of maternal and perinatal morbidity and mortality [1, 2, 20]. Recently, a multidisciplinary, international, consensus-based best practice guideline was provided as a minimum standard of care to minimize associated health risks, and to improve outcomes for pregnant women with skeletal dysplasia [21]. The guidelines concluded, that the increased risks of pregnancy in these situations are related to multiple factors such as cardiopulmonary and musculoskeletal factors in pregnant women. However, all skeletal dysplasias have their own specific features which may affect fertility and obstetric prognosis. Consequently, detailed data on pregnancies in different skeletal dysplasias is warranted.

Our study evaluated pregnancy outcomes in women with CHH from a unique Finnish cohort including 56 women with genetically confirmed CHH. We were able to review a total of 42 pregnancies in 14 women, 26 of which led to delivery. Typical manifestations of CHH, including severe short stature and immunodeficiency, may have negative impact on fertility and reproduction. However, gynecologic and obstetric problems in patients with CHH have received only scant attention. We have previously published reports on diversity of pubertal development and gynecologic health in CHH [11–13]. To the best of our knowledge, no comprehensive obstetric reports have ever been published, and the very limited data on pregnancies in CHH are based on single case reports [14, 15]. This is thus the first study to evaluate reproductive and obstetric outcomes in a larger cohort of women with CHH.

The miscarriage rate in our cohort was 21%, which is not increased compared with rates in the general population (15–25%) [22]. All but one miscarriage occurred during the first trimester. Moreover, 86% (6/7) of women with one or two miscarriages, also had a successful pregnancy and delivery reflecting positive obstetric prognosis. Thus, CHH does not seem to predispose affected women to recurrent miscarriage.

The total number of induced abortions, (12%, 5/42) did not differ from the data for the general population, as reported in the Finnish national pregnancy termination registry [23]. According to the national data, 93% of pregnancy terminations in Finland are performed because of social indications (unplanned pregnancies), compared to only 3.4% for fetal abnormality [23]. In our CHH population, a social indication was reported in 60% (3/5) of the terminations while 40% (2/5) were due to fetal indications. The number of unplanned, terminated pregnancies was thus not increased among the women with CHH. However, the small number of induced abortions does no allow any solid conclusions.

Most of the pregnancies leading to a delivery were full-term. Preterm birth is defined as delivery under 37 completed weeks of gestation. In 2013, in the United States, 11.4% of all babies were born preterm [24]. In Finland in 2018, 5.8% of babies were born under 37 gestational weeks [25]. In our cohort, 96% of babies were born fullterm, and there was only one early preterm birth (pw 25; 1/27 baby born, 3.7%). We did not observe any increase in the rate of complications, such as pregnancy-related hypertensive disorders or gestational diabetes, as compared with the general Finnish and Nordic population [26, 27]. Maternal BMI was increased before pregnancy and an average weight gain during pregnancy was 8.4 kg (18.5 lb). However, according to best-practice guidelines, BMI does not take into consideration body proportions in skeletal dysplasia, and there are no evidence-based recommendations concerning gestational weight gain. A reasonable approximation in short stature women with bone dysplasia is to recommend weight gain in the lowest range, a total of 5–9 kg (11–19.8 lb) over the course of pregnancy [21].

Since the growth of offspring is not generally affected in a recessively inherited disease, normal size of the fetus’ head in a smaller maternal pelvis can cause CPD and result in dystocia. Moreover, breech presentation and malpresentations might be more common in women with short stature. In our study all subjects had a CS, and seven women had recurrent CSs. Recurrent CSs increase the risk for placental abruption, placentation disturbances and post-partum hemorrhage [27]. In our series such complications in women with recurrent CS were not reported. However, a vacuum extraction was required due to CPD and malpresentation twice even in planned CS, highlighting the importance of preparation for intrapartum complications.

The main limitation of this study is the retrospective study design. Number and type of pregnancies and diagnoses during pregnancies were confirmed from national registry. However, information about various subjective symptoms during pregnancy were mainly based on patient interviews and questionnaires. The reliance of self-reported data is subjected to recall bias. This study is by far the largest series reporting outcome of pregnancies in women with CHH. However, as only 14 women altogether were included, the small sample size needs to be acknowledged as a limitation, unavoidable in rare diseases. Phenotypic differences in CHH are wide. Severely affected patients may not try to conceive, they may not conceive spontaneously, or they may not want to participate in the study, and among them obstetric prognosis might be poorer than in this study population. Although we did not systematically collect data on the use of assisted reproductive treatments from the patients, we are aware that these treatments have been used in some instances.

Fertility and reproduction are very important factors in young women’s life. Decision about subjective willingness and capability of carrying a pregnancy should be based on patient´s right to get all potential information preconceptionally. According to best-practice guidelines, preconceptional medical evaluation is recommended to all women with skeletal dysplasia, to consider factors that may impact safety of pregnancy, mode of delivery, and anesthetic management [21]. Due to rarity of skeletal dysplasias, pregnancies need to be assessed and managed in facilities that are aware of the potential complications, and have the skills and resources to anticipate and manage them effectively [21]. Anesthetic assessment should be carried out early in the third trimester because of challenges in anesthetic management and the risk of an emergency CS.

## Conclusions

In conclusion, our study on pregnancies and deliveries in CHH shows encouraging results and indicates that women with CHH, even with remarkable growth restriction, can successfully conceive and complete a normal pregnancy until term delivery. Despite underlying immunodeficiency and increased susceptibility to infections, no infections requiring hospitalization during pregnancy were reported. Because of potential fetal and maternal risks, pregnancies and deliveries in women with CHH warrant careful advance planning and preparing. Further studies, preferably in an international study setting, are needed to further elucidate pregnancy and delivery outcomes in CHH and other rare skeletal dysplasias.

## Data Availability

Restrictions apply to the availability of data generated or analyzed during this study to preserve patient confidentiality. The corresponding author will on request detail the restrictions and any conditions under which access to some data may be provided.

## Abbreviations

CHH: Cartilage-hair hypoplasia
CPD: Cephalo-pelvic disproportion
CS: Cesarean section
IUGR: Intrauterine growth restriction
HILMO: National Hospital Discharge Registry
pw: Pregnancy weeks
BW: Birth weight
BL: Birth length
BP: Blood pressure
